# Cohort protocol: risk assessment of maternal inflammation and early brain development in infants and young children based on multi-source data modeling

**DOI:** 10.3389/fpubh.2025.1530285

**Published:** 2025-07-14

**Authors:** Xianghui Huang, Cuimin Su, Ying Lin, Tianyi Zhou, Ruming Ye, Dan Li, Miaoshuang Liu, Guanhong Wu, Wanting Li, Namei Xie, Xiaofang Deng, Nanxi Zhu, Shaohong Lin, Qin Li, Kai Yan, Deyi Zhuang

**Affiliations:** ^1^Fujian Key Laboratory of Neonatal Diseases, Xiamen, China; ^2^Children's Hospital of Fudan University (Xiamen Branch), Xiamen Children's Hospital, Xiamen, China; ^3^Jinjiang Municipal Hospital, Jinjiang, China; ^4^Department of Maternal and Child Health, School of Public Health, Peking University, Beijing, China; ^5^Children's National Medical Center, Shanghai, China

**Keywords:** maternal inflammation, early brain development, multimodal data, prospective cohort, deep learning

## Abstract

**Introduction:**

Infancy and early childhood are the key stage for the rapid development of brain structure and function, and brain development at this stage has a profound impact on the future intelligence, behavior and health of individuals. A growing body of research suggests that maternal inflammation, as a potential environmental factor, may affect brain development in infants and young children through a variety of mechanisms. Therefore, it is of great significance to evaluate the risk of maternal inflammation to early brain development in infants and young children based on multi-source data modeling to understand the mechanism of early development and prevent brain development disorders.

**Methods and analysis:**

Between December 2021 and May 2024, 360 pairs of pregnant women and their offspring were recruited into the Xiamen Children's Brain Development Cohort. Pregnant women's exposure during pregnancy was collected through standardized and structured questionnaires and medical records. All children were followed up to 3 years of age. We administered questionnaires, behavioral assessments, and performed neuroimaging. Environmental exposures during infancy and early childhood were collected. Children's cognitive, emotional, and linguistic development was evaluated, and blood samples were obtained for whole-exome sequencing and exposure-related biomarker analysis.

**Conclusion:**

In this study, we used deep learning artificial intelligence to construct an early risk assessment model for infant brain development based on the developmental trajectory and developmental results of early brain structure, function, and connections under the complex interaction of “gene-image-environment-behavior” multi-factors, which can improve the early identification and precise intervention of problems in this period, and improve infants cognitive learning and work performance in childhood, adolescence and even adulthood.

**Clinical trial registration:**

https://www.clinicaltrials.gov/; identifier [NCT05040542].

## Introduction

Brain development is currently the most concerned—about frontier field of brain and cognitive science in the world. Brain development during the fetal period is very sensitive to environmental influences. Transcriptional programs in the fetal brain regulate complex developmental processes that ultimately shape the functional structure of the adult brain. Thus, alterations in the intrauterine environment can have far-reaching and long-lasting effects on neurodevelopment (1, 2). Epidemiological data suggest that severe maternal infection during pregnancy is associated with an increased risk of neurodevelopmental and neuropsychiatric disorders, including schizophrenia, bipolar disorder, attention deficit hyperactivity disorder (ADHD), developmental delay, cognitive dysfunction, anxiety/depression, and autism spectrum disorder (ASD), among others (3, 4). In addition, infants experience dramatic changes in brain structure, function, and connections from birth. Synapses are produced in large numbers on the microstructure, and pruning and apoptosis related to the environment and experiences are produced (5); At the macroscopic level, the appearance of magnetic resonance is greatly changed due to the rapid development of axonal myelination. The cortex is significantly thickened, dilated, and folded, and the functional integration is significantly enhanced (6). Long-term follow-up studies have revealed that the cognitive levels reached in infancy and early childhood continue to affect many aspects of physical and mental health, wealth, and longevity in the middle and late adulthood (7). However, currently, our understanding of the mechanisms of these complex brain development processes and the deleterious effects of maternal inflammation on brain development in infants and young children is still limited. Although the micro research carried out in the field of basic neuroscience has reached preliminary conclusions, in order to evaluate the risk factors of brain development and predict the outcome of brain development, it is still necessary to study the brain mechanism of development, especially the map of brain dynamic development, at the macro level. Magnetic Resonance Imaging (MRI), characterized by its non-ionizing radiation and excellent spatial resolution for cerebral tissue, enables the acquisition of multi-parametric information. Furthermore, advanced sequences facilitate functional and biochemical metabolic analyses, thereby providing objective biomarkers for evaluating neonatal brain development and diagnosing cerebral injuries (8). Electroencephalogram (EEG), especially the event-related potential (ERP) technique, can be used to non-invasively explore the cognitive processes of infant speech recognition, language comprehension, phonological awareness, recognition memory, and facial emotion recognition (9, 10). Researchers can, through experimental paradigms specifically designed for different age groups, explore ERP components, characteristics, origins, and influencing factors of language and social-emotional development in infants and toddlers aged 0–3 years (11). These imaging techniques, combined with brain image modeling, can reveal the mysteries of early brain development and the impact of maternal inflammation. Due to the limitations of technology and research objects, it is very difficult to study infant brain development. Under the promotion of the Human Connectome Project (HCP) in the United States, the leading Infant Connectome Project (BCP) has been successfully carried out (12), which has led to a boom in research on infant brain development. Since the 13th Five-Year Plan, China has been taking brain science as a national strategy. Artificial intelligence has been regarded as “a strategic technology to lead this round of scientific and technological revolution and industrial transformation”.

Deep learning technology has made a very important breakthrough in the field of artificial intelligence in the past decade. The availability of big data, the enhancement of computer computing power, and the innovation of deep network training algorithms have greatly promoted the great progress of deep learning technology in many fields, including computer vision, natural language processing, speech recognition, medical image analysis, and disease diagnosis (13–15). In clinical studies, a large amount of multimodal neurobiological information such as neuroimaging, electrophysiological and behavioral data is usually collected from patients, which provide important data support for the early risk assessment of brain development and the prediction of brain development trajectory and results. Deep learning provides effective technical support for mining and understanding the differences and mechanisms of brain development. It is of great scientific significance and application value to analyze, based on deep learning technology, multimodal neurobiological data, develop an accurate and efficient early risk assessment model for brain development, and explore neural biomarkers related to brain development. At present, deep learning networks that are widely used in brain development research mainly include deep belief networks (DBN), convolutional autoencoders (CAE), convolutional neural networks (CNN), graph convolutional networks (GCN), and recurrent neural networks (RNN) (16, 17).

Therefore, this study will establish a 0–3-year-old infants' brain development assessment cohort, collect multimodal neurobiological data, including multi-source data of imaging, EEG, behavior, genes, and environment, and use deep learning artificial intelligence methods to construct an early risk assessment model for infant brain development under maternal inflammation, and carry out individualized comprehensive assessment and prediction of brain intelligence. The aim is to accurately identify and distinguish brain development disorders in infants and young children, and to discover the neural mechanism in the brain network based on the proposed interpretable artificial intelligence method, to propose a new method for the clinical assisted diagnosis and assisted rehabilitation of brain development.

## Objectives

1. Establish a brain development assessment cohort for infants and young children aged 0–3 years, and collect multimodal data of “gene-image-environment-behavior” through high consistency, high success rate, high follow-up rate and high quality.
2. Use MRI and EEG and image recognition technology to depict the early development trajectory of brain structure, function and connection of these high-level functions.
3. Integrate multi-source data: use deep learning artificial intelligence methods to construct an early risk assessment model for infant brain development under maternal inflammation, and conduct individualized comprehensive assessment and prediction of brain intelligence.
4. Integrate whole-exome sequencing and whole-genome sequencing results to identify genes that play a key role in children's early life development. Also, identify how these genes interact with maternal inflammation to affect children's brain structure and function development.

## Methods and analysis

### Research subject

The study subjects were infants and children aged 0–3 years and their mothers, and the Xiamen Children's Brain Development Cohort recruited the study subjects from the society (kindergartens, elementary schools, and other institutions) and the neonatal department of Xiamen Children's Hospital. Based on the information of the mothers' pregnancy—related medical history, infants whose mothers had high inflammatory indices during pregnancy (e.g., CRP, interleukin−1β, interleukin−6, interleukin−10, and macrophage inflammatory factor−1β), or had chorioamnionitis, pathologic abnormalities of the placenta, preterm premature rupture of the membranes, or had combined gestational hypertension and diabetes mellitus with maternal inflammatory diseases were included in the observation group. Infants without maternal inflammation were the control group.

Characteristics of our included subjects included:(1) age 0–3 years;(2) gestational age 37–42 weeks;(3) birth weight >2.5 kg; and(4) no significant birth complications. Characteristics of excluded subjects included:(1) history of resuscitation from asphyxia at birth;(2) congenital structural anomalies;(3) presence of inborn inherited metabolic disorders such as phenylketonuria, galactosemia, and congenital hypothyroidism;(4) presence of a major medical condition affecting growth, or development; and(5) contraindications to magnetic resonance imaging (MRI).

#### Sample size

The formula for calculating the sample size is based on the comparison of the average values of the two independent samples:


$$\begin{array}{l} N\;=\;\frac{{2\left(Z_{\alpha }+Z_{\beta }\right)}^{2}\sigma^{2}}{d^{2}} \end{array}$$

Where σ is the estimated standard deviation, *d* represents the difference between the two sample means, and *Z*_α_ and *Z*_β_ are the quantiles of the standard normal distribution corresponding to α and β, respectively. A previous Greek study using the McCarthy Scales for Children's Abilities to measure children's brain development found that the difference between the memory scores of children born to mothers with a hyperinflammatory state during pregnancy and those born to normal mothers was −4.4 (−8.3, −0.5) (18). Substituting σ = 0.05 and β = 0.2 gives *N* = 1603, based on a 1:1 calculation of mothers in the inflammatory group vs. the normal group, a total of 2*N* = 3206 pregnant women needed to be included in the entire study.

Hospital clinicians and nurses explained the objectives of the study, the process, the potential benefits of participation, and the confidentiality of the study to all families eligible for inclusion. All investigators entering the cohort signed an informed consent form. The Xiamen Child Brain Development Cohort provided each mother and child pair with a unique family ID for further follow-up. Participants could withdraw from the cohort at any time. The study was approved by the Clinical Ethics Committee of Xiamen Children's Hospital and the participating hospitals.

#### Follow-up plan

**Figure 1:**
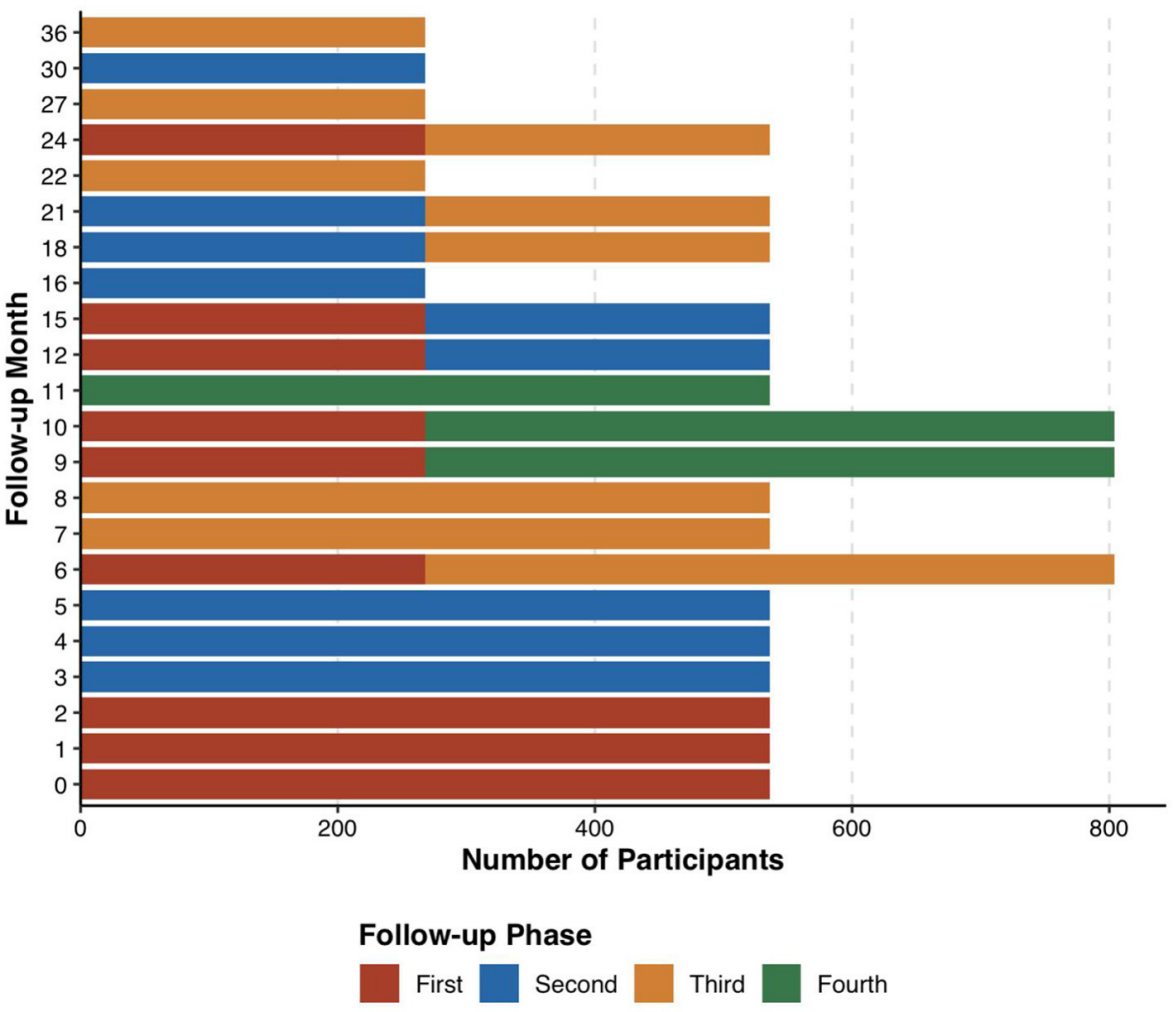
Aggregated cohort follow-up time planning schedule.

Utilizing an aggregation cross design, referencing the grouping methodology of the Baby Connectome Project (BCP) by UNC/UMN in the USA (12), participants were categorized into 9 sub-cohorts based on the age at initial enrollment, each having varying sampling density and age spans (Table 1). Each of the first three sub-cohorts enrolled 536 participants, while sub-cohorts 4–9 each enrolled 268 participants, totaling 3,216 participants. Each participant was followed up 3–4 times, yielding data from 11,256 participant—visits. Moreover, the follow-up intervals for sub-cohorts 1–3 and 4–9 were 3 and 6 months, respectively, ensuring sufficient sampling density during the first year, which is critical for rapid development in language and socio-emotional skills. Oversampling was strategically implemented during milestones of language and socio-emotional development and periods prone to scan failures (before the age of 3). Considering the low cooperation levels of infants and potential dropouts, 3,847 participants were recruited to ensure the acquisition of high-quality data from 3,216 subjects (Figure 1).

**Table 1:** Cohort design and follow-up time planning schedule.

**Aggregated cross design**						
**Cohort number**	**Number**	**Follow-up**				**Note**
		**First**	**Second**	**Third**	**Fourth**	
1	536	0	3	6	9	High-density sampling: once every 3 months
2	536	1	4	7	10	
3	536	2	5	8	11	
4	268	10	16	22		Medium-density sampling: every 6 months
5	268	6	12	18		
6	268	9	15	21		
7	268	12	18	24		
8	268	15	21	27		
9	268	24	30	36		
Total	3,216	Number of Participant—visits: 11,256			
Target number	3,847	Considering the failure rate of magnetic resonance imaging and the dropout rate of the cohort, the effective enrollment was guaranteed to be >11,256 cases				

The study protocol encompasses questionnaires, behavioral assessments, and brain structure and function examinations from the perinatal period of children through the age of 3 years, complemented by whole-exome sequencing. Basic information about the pregnant women and their partners was retrospectively collected at the time of enrollment.

The items and contents of the data collected at the time of entry and at different follow-up age points are detailed in Table 2 (Detailed descriptions of the scales and tasks used during the follow-up visits can be found in the Supplementary material). The study collects brain images, EEG data, data about the environment, and data about the genes of infants and young children, and evaluate cognitive development, emotional development, and language development of infants and young children.

**Table 2:** Timeline for follow-up programs by age group.

**Test content**	**Tool name**	**Age group (month)**						
		**Perinatal period**	**0–1**	**2–6**	**7–12**	**13–18**	**19–24**	**25–36**
**Brain structure and function**								
Brain MRI	/		√	√	√	√	√	√
**Electrophysiological monitoring of brain waves**								
EEG	/		√	√	√	√	√	√
**Behavioral assessments**								
Developmental level	Griffiths Development Scales-Chinese Edition (GDSC) (19)		√	√	√	√	√	
Intelligence	Wechsler Intelligence Scale for Children—Fourth Edition (WPPSI-IV) (20)							√
**Maternal and Parenting Issues—Parental Questionnaire**								
Basic Information	Background questionnaire	√	√	√	√	√	√	√
Family parenting environment	Index of Child Care Environment (ICCE) (21)					√	√	√
Parenting Stress	Parenting Stress Index-Short Form (PSI-SF) (22)		√	√	√	√	√	√
Childbirth experience	Postpartum Experiences Questionnaire within 3 months (PPQ) (23)		√	√				
Work-family conflict	Work-Family Conflict Scale (24)		√	√	√	√	√	√
Pregnancy-Life events	Pregnancy Life Events Questionnaire (PLQ) (25)	√						
Pregnancy stress	Pregnancy Stress Scale (PPS) (26)	√						
Depression	Self-Rating Depression Scale (SDS) (27)		√	√	√	√	√	√
Anxiety	State-Trait Anxiety Inventory for Young Adults (STAI-Y) (28)		√	√	√	√	√	√
Sleep quality	Pittsburgh Sleep Quality Index (PSQI) (29)	√						
Postpartum depression	Edinburgh Postnatal Depression Scale (EPDS) (30)		√	√	√			
Breastfeeding Self-Efficacy	Breastfeeding Self-Efficacy Scale-Short Form (BSES-SF) (31)		√	√				
Mother-infant bonding	Maternal-Infant Bonding Scale (MIBS) (32)				√			
Mother-infant attachment	Maternal Postnatal Attachment Scale (MPAS) (33)		√	√	√			
Parenting style	Parenting Styles and Dimensions Questionnaire (PSDQ) (34)							√
Family functioning	Family Assessment Device (FAD) (35)		√					
Collaborative parenting	Co-Parenting Questionnaire (36)							√
**Children Issues—Parental Questionnaire**								
Baby sleep	Brief Infant Sleep Questionnaire (BISQ) (37)		√	√	√	√	√	√
Childlike temperament	Chinese Toddler Temperament Scale (CTTS) (38)					√	√	√
Social emotions	Ages & Stages Questionnaires: Social-Emotional, 2nd Edition (ASQ:SE-2) (39)		√	√	√	√	√	√
Language development	Putonghua Communicative Development Inventory-Words and Gestures“ (PCDI: Words and Gestures) (40)				√	√		
	Chinese Communicative Development Inventories: Words and Sentences” (CCDI: Words and Sentences) (41)						√	√
Behavioral assessment	Child Behavior Checklist (CBCL) (42)					√	√	√
Autism spectrum disorder screening	Modified Checklist for Autism in Toddlers, Revised with Follow-up(The M-CHAT-R/F) (43)					√	√	√
Use of electronic devices	Seven-in-Seven Screen Exposure Questionnaire (44)						√	√
**Behavioral experiments**								
Psychological theory	Theory of Mind (ToM) (45)							√
Executive function/self-control	Head Toes Knees Shoulders (HTKS) (46)							√
Emotion-related self-regulation, etc	Emotional Stroop Task (47)							√
**Behavioral observations**								
Negative emotion regulation, social emotion	Still Face Experiment (48)			√	√			
Language expresses social emotions	Mother–Infant Interaction (49)			√	√	√		
Executive control	Strange Situation Test (50)						√	
Executive control	Frustration Tolerance Task (51) + Delay of Gratification Task (52)							√
**Blood**								
Genetic and exposome	/	√						

#### Brain imaging data

At each follow-up visit, the data of the brain magnetic resonance examination in the non-sedated state of the subjects were collected, including skull structure MRI (sMRI), resting-state fMRI, dMRI sequence and multi-parametric MRI (MTP) scans for a total of 40 min. All MRI data were acquired on a 3T scanner (uMR 890, United Imaging) with a 64-channel head coil. The scanning protocol was as follows: Structural MRI: High-resolution T1w/T2w images (0.8 mm isotropic) using AI-assisted compressed sensing (ACS) (53), achieving 37.8% time reduction compared to BCP protocols (5). Resting-state fMRI: Single-shot EPI (TR = 800 ms, 1.8 mm isotropic) with multiband factor = 8 and field-map-based distortion correction. Diffusion MRI: 72 directions with 3 shells (*b* = 500/1,000/3,000) and dual phase-encoding, utilizing incremental acquisition for sampling uniformity (54). Multi-parametric MRI (MTP): Gradient-echo sequences generating PDw/T1w/T2w images and quantitative maps (PD/T1/T2/QSM) through dual-TR/dual-flip-angle design. Automated slice positioning (Easy Scan) was used to standardize scan planning. For sleeping subjects, supplemental scans (fMRI with reversed phase-encoding; dMRI at *b* = 1,500/2,000/2,500) were added to mitigate geometric distortions. The detailed MRI acquisition protocol is summarized in Table 3.

**Table 3:** The parameter list of the multi-MRI protocol (12, 54, 55).

**Sequence**		**Matrix**	**FoV (mm)**	**Resolution (mm)**	**TE (msec)**	**TR (msec)**	**Slices/orientation**	**Time (min:sec)**	**Acceleration strategy**
Localizer		115 × 144	300 × 300	2.6 × 2.08 × 4	1	2.7	1/Sag	00:10	-
T1-weighted		320 × 300	256 × 240	0.8 × 0.8 × 0.8	2.9	7.4	208/Sag	03:44	ACS, × 3.3
T2-weighted		320 × 300	256 × 256	0.8 × 0.8 × 0.8	452	3,000	208/Sag	04:09	ACS, × 3.8
Field Map		116 × 116	209 × 209	1.8 × 1.8 × 1.8	2.46	501.2	72/Axial	00:58	-
BOLD Rest Ref PA		116 × 116	209 × 209	1.8 × 1.8 × 1.8	35	800	72/Axial	00:25	Multi-band = 1
BOLD Rest PA		116 × 116	209 × 209	1.8 × 1.8 × 1.8	35	800	72/Axial	06:25	Multi-band = 8
DWI b0 AP		140 × 140	210 × 210	1.5 × 1.5 × 1.5	77.5	3,000	92/Axial	00:40	Multi-band = 4
DWI	PA, *b* = 500, 1,000, 3,000	140 × 140	210 × 210	1.5 × 1.5 × 1.5	77.5	3,014	92/Axial	04:29	Multi-band = 4, 3 shells, 72 directions
	AP, *b* = 1,500, 2,000, 2,500	140 × 140	210 × 210	1.5 × 1.5 × 1.5	77.5	3,014	92/Axial	04:29	
MTP		192 × 192	230 × 190	0.8 × 0.8 × 1.6	20.76	36.8	70/Axial	7:10	ACS, × 4
**Contingent on the baby continuing to sleep**									
BOLD Rest Ref AP		116 × 116	209 × 209	1.8 × 1.8 × 1.8	35	800	72/Axial	00:25	Multi-band = 1
BOLD Rest AP		116 × 116	209 × 209	1.8 × 1.8 × 1.8	35	800	72/Axial	06:25	Multi-band = 8
DWI	PA, *b* = 500, 1,000, 3,000	140 × 140	210 × 210	1.5 × 1.5 × 1.5	77.5	3,014	92/Axial	04:29	Multi-band = 4, 3 shells, 72 directions
	AP, *b* = 1,500, 2,000, 2,500	140 × 140	210 × 210	1.5 × 1.5 × 1.5	77.5	3,014	92/Axial	04:29	

#### EEG examination

EEG data were collected at each follow-up visit. EEG recordings are conducted using a 32-channel actiCAP active electrode system (Brain Products GmbH) with GreenTek GT5 conductive gel, positioned according to the standardized 10-10 international system (56). Signals are amplified via a BP actiCHamp Plus amplifier and monitored in real-time using BrainVision Recorder software. Electrode impedances are maintained below 5 kΩ throughout sessions via automated DRT quality control software (MATLAB R2018a runtime environment). All infants (0–3 years) are tested in naturally alert states, verified through continuous behavioral observation. One operator manages device calibration and event marking, while a second observer documents infant behaviors (e.g., head movements, vocalizations) .

The EEG Experimental Tasks Include:

(1) Resting State (Resting): The test involves the acquisition of a 5-min resting state.
(2) Three Language Tasks: (1) Infant Speech Perception Ability (FFR/Oddball): The test involves presenting two types of auditory stimuli in a pseudo-random manner. (2) Infant Vocabulary Semantic Development in Social Scenarios (SceneWord): The test involves observing infants' responses to auditory stimuli after they view pictures with situational and environmental information. (3) Development of Infant Syntactic Ability (Syntax): The test content is the continuous input of auditory stimuli in a regular pattern.
(3) Two Emotion Tasks: (1) Emotion Classification (Face Emotion): The test involves rapidly presenting images or stimuli representing different emotions. (2) Empathy with Natural Stimuli (Empathy): The test requires the infants to watch a silent animated cartoon.

To ensure that sufficient data can be collected for this task modality, the research team has decided to adopt a rotation method based on the age of the participants and the categories of the multimodal tasks. The rotation requirement is that in each experiment, 2 tasks are selected from the language tasks, 1 task is selected from the emotion tasks, and the resting state task, which is mandatory, is added. In total, there are 4 tasks. The estimated duration of the tasks is ~30–40 min.

#### Behavioral assessment

(1) Griffiths Development Scales—Chinese Edition (GDSC) (57): Griffith assessment was performed during the follow-up of preterm infants after discharge. Griffiths scale is a standardized assessment scale for children aged 0–8 years whose mother tongue is Chinese and which has Chinese norms. The scale is divided into two parts: 0–2 years old and 0–8 years old, the 0–2 years old part is composed of 5 domains: “A Movement”, “B Individual-Society”, “C Language”, “D Hand-eye Coordination” and “E Performance”, and the 0–8 years old part adds “F Practical Reasoning Domain” on this basis. When the developmental quotient DQ < 70, it indicates developmental delay; when DQ ≥85, it indicates normal development.
(2) Wechsler assessment: this study uses the WPPSI—IV (58) to assess the child's intelligence level. Five scores are calculated from the questionnaire, namely the Verbal Comprehension Index (VCI), Visual Spatial Index (VSI), Working Memory Index (WMI), Fluid Reasoning Index (FRI), and Processing Speed Index (PSI). Then, the scores of different ability areas will be summarized based on the questionnaire data to obtain the Full Scale IQ (FSIQ) score, which comprehensively reflects the overall intellectual level of children. Different intellectual level intervals are divided according to the FSIQ.

#### Parent-questionnaire

An electronic questionnaire was designed on the WeChat public account platform. After parents follow and register, the system will push the parent questionnaire of the corresponding age group for parents to fill in. The electronic questionnaires were divided into two main categories: maternal and parenting issues, and children's issues, as detailed in Table 2.

(1) The Maternal and Parenting Issues Questionnaire primarily investigates the family parenting environment , maternal parenting stress, pregnancy and postpartum events, postpartum emotional state of mother and work-family relationships. It further explores the impact of these environmental factors on children's brain development.

Among them, the Family Parenting Environment Survey uses the Index of Child Care Environment (ICCE) (21): the scale has a total of 13 questions and includes family interaction, external contact, avoiding scolding and social support. The total score of the scale ranges from 0 to 13 points, and the higher the score, the better the child's nurturing environment. According to the distribution of population scores, the ICCE total score was divided into four groups: the worst family environment group (≤ 10 score), the lower-middle group (11 score), the upper middle group (12 score), and the best group (13 score).

(2) The Children's Related Issues Questionnaire mainly investigates the sleep of infants, childlike temperament, social emotions, language development, behavioral assessment, autism spectrum disorder screening and use of electronic devices. Among them, (1) the Ages and Stages Questionnaires-Social-Emotional (the 2nd edition) (39), focuses on the emotional development and mental health of infants and young children. There are two types of screening results for ASQ—SE: below the boundary value and equal to or higher than the boundary value. The latter indicates that the child's development is in line with his or her monthly development level. (2) Language development: a. Infant and child communication development questionnaire: vocabulary and gesture short form. This scale was jointly revised by the University of Michigan, the National University of Cork, Ireland, and Peking University in 2008. The vocabulary scale of the scale has a total of 219 items, which contains most of the vocabulary used by normal Chinese children, such as: person, food, animal, drink, body, parts, as well as verbs, adverbs, quantifiers, and tenses. The “Vocabulary and Gesture Short Table” examines the items that children “don't understand”, “understand”, and “can speak”. “don't understand” gets 0 points, and “understand” and “can speak” get 1 point each. b. The Children's Communication Development Questionnaire: Vocabulary and Sentence Short Tables. It examines items of whether children “can't speak” or “can speak” and “can speak” items, “Can't speak” gets 0 points, “Can speak” gets 1 point.
(3) The M-CHAT-R/F (43) (Modified Checklist for Autism in Toddlers, Revised with Follow-up) is a screening tool used to assess the risk of autism spectrum disorder in toddlers aged 16 −30 months. The screening process consists of two steps: the first step is an initial questionnaire, and the second step is a follow-up interview. The questionnaire is completed by the child's parents or main caregivers based on the child's actual behavior.

#### Behavioral experiments

(1) Psychology Theory Task (45) (36 months of age and above)—tests the child's psychotheoretical ability.
(2) Head, Foot, Knee, and Shoulder (HTKS) Task (46) (25-month-old and older)—tests the child's executive function/self-control.
(3) Emotional Stroop Task (47) (25-month-old and older)—tests children's ability to self-regulate emotionally.

#### Behavioral observations

(1) Still Face (48) (3–12 months old)—This task is used to test the negative emotion regulation and social emotion of infants. It consists of three stages: face-to-face interaction, still-face episode, and reunion. Measuring infants' ability to achieve interactions by regulating emotional expression reflects infants' early goal—oriented social competence.
(2) Mother–Infant Interaction (49) (6–18 months of age)—This task tests for verbal expression and social emotion. This semi-structured observation records 10 min of mother–child interaction. During the observation, parents are encouraged to interact with their children in a normal way. This observational experiment can reflect a number of indicators, such as maternal sensitivity, infant social referencing, dyadic contingent responses, etc.
(3) Strange Situational Test (50) (21–27 months old)—Inhibition/Control Ability: Three strangers interacted with children in the presence and absence of their mothers, respectively, to assess the infant's attachment security.
(4) Frustration Tolerance & Delayed Gratification Task (51) (30–42 months old)—To test inhibition/control ability: The first paradigm was adapted from the classic marshmallow experiment, using snacks and crayons to observe children's self-control. The second paradigm presents the child with a situation in which the goal is blocked. In these tests, toddlers' patience time, coping strategies, and parental responses will be classified and coded.

#### Genetic and exposome testing

At the time of enrollment, 2 ml of blood from each parent and child were drawn once, whole exome sequencing and exposome were performed, and multivariate statistical methods were used to analyze genes and biomarkers related to brain, early language and emotional development. In addition, through the deep sequencing of the whole genome, a variety of genetic analysis methods, including candidate-gene analysis, whole-gene analysis, and multi-gene risk combination analysis methods, will be used to explore the molecular mechanisms of brain structure, function, connections, and the developmental trajectory of language and emotion, and elucidate the precise mechanism of gene expression during human brain development.

### Outcomes

#### Primary outcome

##### Cohort and data goals

This study will create a 0–3-year-old infant brain development assessment cohort. This study aims to gather comprehensive “gene – environment - image - behavior” multimodal data, with a focus on high-quality collection. The data will be used to establish a solid foundation for research on infants brain development, and the data integrity rate is expected to be at least 90% across all data categories.

##### Brain development mapping

Using advanced magnetic resonance imaging (MRI) and electroencephalogram (EEG) technologies, we will precisely map the early brain development of infants and determine the developmental patterns of infant brain functions. This includes determining, through technologies such as image recognition, the brain development curves of infants with age, as well as the normal volume ranges of different brain regions at each age group. It also involves recording the growth patterns of brain structures such as the hippocampus and prefrontal cortex, and the development of neural connections. By analyzing the data, we will map the brain development trajectories of Chinese children and identify the critical periods of brain development.

##### Risk model construction

Using deep-learning-based artificial intelligence technology, we will develop an early risk assessment model for the impact of maternal inflammation on infant brain development. This model will be able to integrate data on maternal inflammation during pregnancy to predict the brain development of children with high precision and further identify potential risks in infant intelligence, motor skills, emotions, and cognition. Through rigorous training and validation, our goal is to ensure that the area under the curve (AUC) of the model's recognition ability for relevant developmental disorders reaches 0.8 or above.

##### Gene-environment interaction

Integrate the results of whole-exome sequencing to identify genes crucial for early-life brain development. Through in-depth genetic and statistical analyses, this study will explore how these genes interact with maternal inflammation and environmental exposure during early life. This study anticipates uncovering at least 10 genes that show significant interaction effects and elucidating the underlying molecular mechanisms.

#### Secondary outcome

In the secondary results of this study, we will mainly conduct an in-depth exploration of the impact of environmental factors on children's brain development from two aspects, namely the maternal environmental exposure during pregnancy and the family environment and parenting patterns after birth.

##### Research plan on the impact of maternal adverse factor exposure during pregnancy on infant early brain development

Focusing on maternal exposure to inflammation during pregnancy, this plan aims to analyze its mechanism of action on infant early brain development. On the one hand, we will study the correlation between the changes in inflammatory indicators during pregnancy and infant brain development. By comparing the brain imaging and electrophysiological data of children under different inflammatory levels, as well as the development of children's intelligence, cognitive ability, motor ability and other abilities, we will explore the potential impact of maternal inflammation during pregnancy on the development of brain structure and function. On the other hand, regarding other adverse conditions during pregnancy, such as chorioamnionitis, placental pathological abnormalities, gestational diabetes mellitus, gestational hypertension and other pregnancy-related diseases, as well as adverse environmental exposure during pregnancy, we will analyze their impacts on the structure and function of infant brains, and the potential links between such impacts and the future development of infants in terms of intelligence, cognition, emotion, and motor skills.

##### Research plan on the impact of family environment and parenting patterns after birth on infant brain development

This study will conduct research on the family environment and parenting patterns after birth, and analyze their impacts on the structure and function of infant brains. With the help of professional assessment scales, this study will explore the relationship between family parenting patterns and infant brain development, paying particular attention to the promoting or inhibiting effects on intellectual development and the underlying pathways. At the same time, this study will examine the impact of the family environment on infant emotional regulation ability, as well as the differences in infant cognitive development under different parenting styles, and further explore the potential neural mechanisms through which these factors affect infant brain functions.

#### Data analysis

##### Cohort and data objectives

Conduct an integrity assessment of the “gene-environment-electroencephalogram (EEG)—imaging-behavior” multimodal data for the included cohort of infants and toddlers aged 0–3 years. Calculate the integrity of core data such as questionnaire data, gene data, and children's behavior data, with an integrity rate of at least 90%. For data with high noise levels such as brain magnetic resonance imaging (MRI) and EEG, the integrity rate should be ≥85%, and the overall integrated integrity should be ≥80%. Examine the reasons for missing data. For completely random missing data of different types, use methods such as multiple imputation, k-nearest neighbors (KNN) imputation, the Generative Adversarial Imputation Network (GAIN) algorithm, and expert judgment filling to supplement the data. Filter gene data through the Genome Analysis Toolkit (GATK), annotate it with ANNOVAR, and screen high-quality loci through the Hardy-Weinberg Equilibrium (HWE) test. Remove outliers from environmental data regarding inflammatory indicators during pregnancy, pregnancy complications, and family parenting scales. Process MRI structural images using Free Surfer/FSL and functional magnetic resonance imaging (fMRI) using Statistical Parametric Mapping (SPM) for imaging data. Preprocess EEG data using EEGLAB. Transform behavior data into Z-scores. Present demographic characteristics through descriptive statistics. Use the chi-square test/analysis of variance to compare baseline differences among subgroups such as gender and gestational age to ensure the balance of the cohort and lay the foundation for subsequent analyses.

##### Mapping of brain development atlas

Based on the preprocessed MRI and EEG data, we use FreeSurfer to quantify the volumes of brain regions such as the hippocampus and prefrontal cortex, and calculate the normal range of brain region volumes related to age. Calculate and plot the curves of the volumes of specific brain regions of children by gender through non-linear functions as they change with age, and identify the critical windows of development for specific brain regions in infants. Extract time-frequency features such as α/β wave power from EEG data, construct a functional connectivity matrix, and analyze the development pattern of the resting-state EEG network through graph theory analysis and independent component analysis. Use canonical correlation analysis to explore the coordinated development relationship between the volume of MRI brain regions and the functional connectivity of EEG. At the same time, conduct piecewise regression on longitudinal data, detect the mutation points of the brain development rate, and verify its correlation with the development scores in terms of intelligence, cognition, and behavior, so as to comprehensively depict the dynamic development trajectory of brain structure and function.

##### Construction of infant brain development models

Integrate the volumes of MRI brain regions, time-frequency features of EEG, inflammatory indicators during pregnancy, and questionnaire scores of children in different ability areas. Reduce the dimensionality through principal component analysis to generate multimodal feature vectors and input them into a Transformer to construct a deep learning model. Train the model using cross-validation and prevent overfitting through the Dropout method and the AdamW optimizer. Take the AUC as the core index, and simultaneously calculate the sensitivity, specificity, accuracy, and other indicators of the model. Verify the generalization ability of the model using an international public database, and locate the key predictive factors through the SHapley Additive exPlanations (SHAP) values and the Local Interpretable Model-agnostic Explanations (LIME) algorithm. Combine the anatomical localization of brain regions to explain the risk prediction mechanism of how inflammation during pregnancy affects brain development, and ensure the clinical application value of the model.

##### Gene-environment interaction

In the data analysis of gene–environment interactions, this study will be base on whole exome sequencing data. First, variant detection and quality control will be performed using the Genome Analysis Toolkit (GATK). ANNOVAR will be utilized for functional annotation of the variants, and high-quality loci will be screened through the Hardy-Weinberg Equilibrium (HWE) test. We will focus on the variants of candidate genes related to brain development and loss-of-function mutations. Subsequently, the Sequence Kernel Association Test with Optimal weighting (SKAT-O) will be employed to analyze the association between gene sets and brain development phenotypes. Meanwhile, a generalized linear model will be constructed, incorporating pregnancy inflammation indicators (such as CRP and IL-6 levels), environmental exposure variables, and their interaction terms with gene variants to test the gene–environment interaction effects. By referring to the literature in fields such as neural synapse development and inflammatory signaling pathways, genes with significant effects in the interaction will be screened out. For the screened genes, methods such as metabolomics and proteomics will be further used to verify their regulatory mechanisms in brain development, analyze the impact of genes on the early-life brain structure and function of infants, and ultimately reveal the molecular pathways through which the interaction of genes, pregnancy inflammation, and environmental exposure affects early brain development. It is expected to identify at least 10 key genes with significant effects and clarify their potential biological mechanisms.

##### The impact of family environment and parenting patterns on brain development

Regarding the secondary research results, we will conduct a comprehensive analysis from two aspects: the exposure of adverse factors in mothers during pregnancy and the family environment and parenting patterns after birth. For maternal exposure to adverse factors during pregnancy, on the one hand, regarding the correlation between inflammatory indicators and brain development, we will conduct correlation analysis between brain indicators such as MRI-derived brain volume, cortical thickness, and EEG power spectral density, and the levels of inflammatory cytokines such as C-reactive protein (CRP) and Interleukin-6 (IL-6). Group children according to the inflammatory levels and compare their brain imaging, electrophysiological data, and scores of relevant abilities of children. On the other hand, for other adverse conditions during pregnancy, conduct regression analysis to explore the potential links with the future development of children. In the analysis of family factors after birth, first conduct correlation and regression analysis between the scores of the quantified family parenting scale and brain indicators such as MRI-derived brain volume, cortical thickness, and EEG power spectral density. At the same time, explore the pathways through which parenting patterns affect children's intellectual development through mediation analysis. In addition, associate family environment variables with children's emotional regulation indicators, and compare the differences in children's cognitive development under different parenting styles.

## Preliminary results

Between December 2021 and May 2024, 360 pairs of pregnant women and their offspring were recruited into the Xiamen Childhood Brain Development Cohort, which investigated basic family information through a baseline questionnaire, children's births through medical information, and MRI scans of children's brains who entered the cohort. Of the 360 children enrolled in the study (Table 4) , 203 (56.39%) were males, the mean gestational week of the mother's pregnancy was 35.67 weeks, the mean age of the children at MRI was 3.28 weeks, and there were a total of 163 (45.3%) children born at term and 156 (43.3%) children born by normal delivery.

**Table 4:** Baseline characteristics and risk factors of research subjects.

**Variable**	**Sum mean (sd)**	**Male mean (sd)**	**Female mean (sd)**	***p***
People (%)	360	203 (56.39)	157 (43.61)	
Gestational age (week)	35.67 (3.88)	35.85 (4.03)	35.45 (3.68)	0.332
Age at scanning (week)	3.28 (3.30)	3.25 (3.49)	3.31 (3.05)	0.849
Full-term infant (%)	163 (45.3)	102 (50.2)	61 (38.9)	0.041
Birth weight (g)	2.49 (0.87)	2.58 (0.87)	2.37 (0.85)	0.024
Vaginal delivery (%)	156 (43.3)	97 (47.8)	59 (37.6)	0.067
Parity (%)				0.141
0	55 (15.3)	30 (14.8)	25 (15.9)	
1	163 (45.3)	84 (41.4)	79 (50.3)	
≥2	142 (39.4)	89 (43.8)	53 (33.8)	
Chorioamnionitis (%)	7 (1.9)	4 (2.0)	3 (1.9)	1.000
Gestational hypertension (%)	40 (11.1)	18 (8.9)	22 (14.0)	0.170
Gestational diabetes (%)	76 (21.1)	38 (18.7)	38 (24.2)	0.257
Pathological placental abnormalities (%)	7 (1.9)	2 (1.0)	5 (3.2)	0.265
Premature rupture of membranes (%)	47 (13.1)	25 (12.3)	22 (14.0)	0.752
Eclampsia (%)	27 (7.5)	13 (6.4)	14 (8.9)	0.486
Sexually transmitted diseases (%)	28 (7.8)	15 (7.4)	13 (8.3)	0.909
Antibiotic treatment (%)	68 (18.9)	34 (16.7)	34 (21.7)	0.297
Maternal inflammatory (%)	187 (51.9)	94 (46.3)	93 (59.2)	0.020

## Discussion

**Figure 2:**
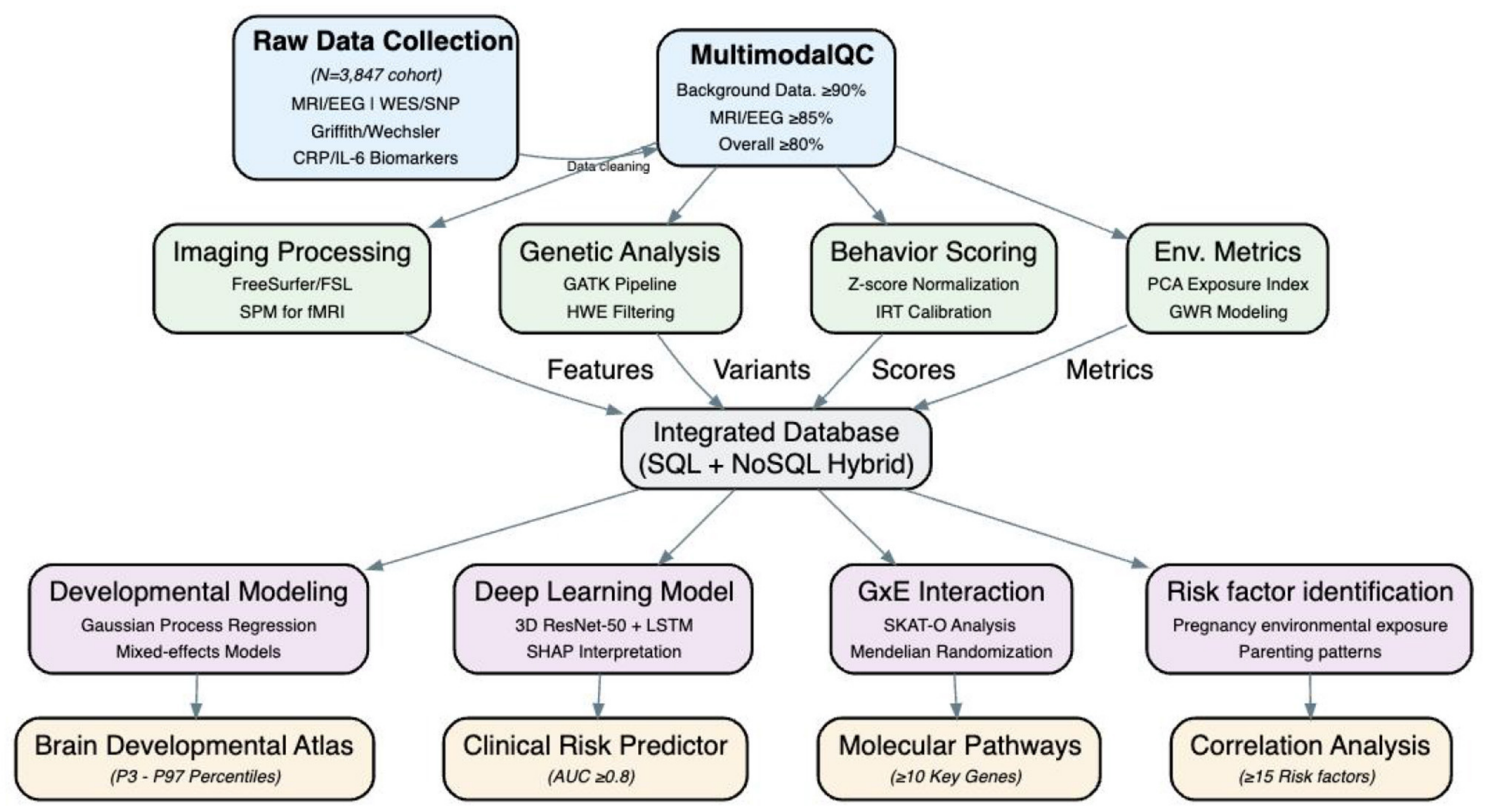
Technology roadmap for the full research program.

The aim of this study is to establish a cohort for evaluating the brain development of infants aged 0-3 years (Figure 2). It involves collecting MRI, EEG, and other data in the early life of infants. Through image segmentation, feature extraction, and functional connectivity analysis techniques, it aims to depict the early development trajectories of brain structure, function, and infant brain development. Meanwhile, environmental risk factors in the early life of infants that affect brain development will be identified. Results from whole-exome sequencing will be integrated to analyze which genes play a crucial role in the early brain development of children. In addition, analyses will be conducted to explore how genes interact with environmental risk factors and influence the structural and functional development of infants' brains. Deep-learning algorithms will be employed to develop an early-risk assessment model for infant brain development, enabling individualized comprehensive assessments and predictions of brain intelligence.

### Construction of high-precision brain function map

This study will construct a map of brain function and structure from birth to early childhood and capture the dynamic changes in brain structure and function through multimodal neuroimaging techniques, including MRI and EEG. MRI will be particularly useful for visualizing structural changes, while EEG will provide insights into functional connectivity and electrical activity. Time series analysis methods will be applied, processing sequential imaging data collected from birth to early childhood to reveal key time nodes and developmental pathways in brain development. We will develop and optimize image processing algorithms, specifically for the particularity and complexity of infant brain imaging data, to ensure the accuracy and effectiveness of data analysis.

### A comprehensive analysis of the interaction between genes and the environment

In addition, the aim of this study is to analyze in depth the association between maternal inflammation and the risk of early brain development in infants and the role of genetic factors in this association, as well as to explore the risk factors for child neurodevelopment. The study will utilize whole exome sequencing technologies combined with detailed data on children's exposures (including maternal inflammation during pregnancy, nutrition, home environment, social interactions, exposome data, etc.) to analyze the interaction between genetic and environmental factors through statistical modeling. In the process, genetic information and environmental data will be integrated and analyzed, and the impact of environmental factors will be assessed to provide a comprehensive understanding of how environmental exposures during pregnancy and genetic information influence early childhood neurodevelopment.

### Early risk assessment and prediction model development

**Figure 3:**
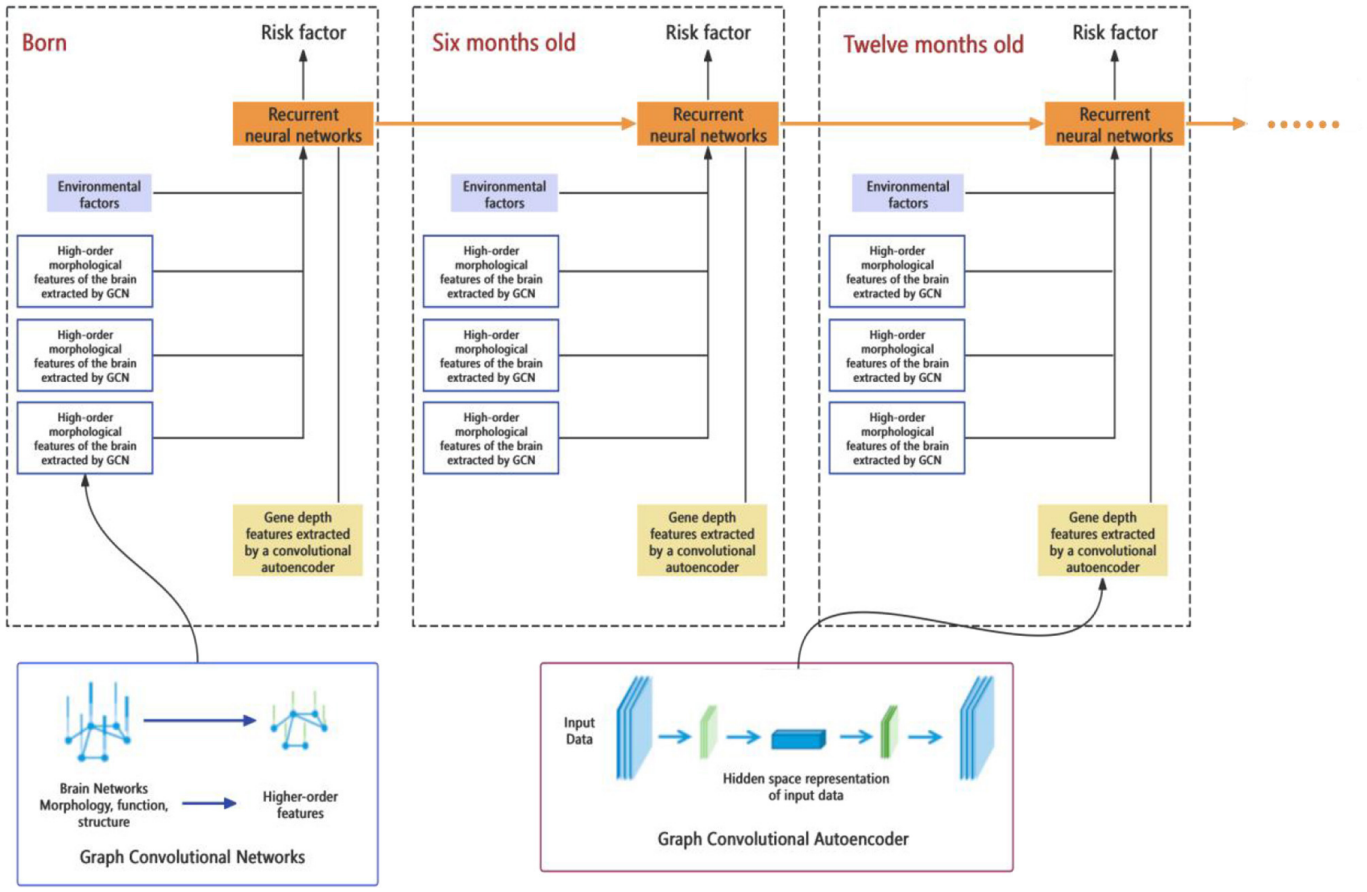
Deep learning model for early risk assessment of infant brain development.

This part of the study aims to develop a comprehensive predictive model for early brain developmental disorders, based on multi-source data from neuroimaging, genetics, environmental exposures, etc. Machine learning and artificial intelligence technologies (such as deep learning, support vector machines, etc.) are applied to analyze this data and build predictive models for early risk assessment (Figure 3). Finally, based on these models, an easy-to-use personalized risk assessment tool will be developed to provide clinicians and researchers with precise early intervention and diagnostic support.

### Strength

(1) High-standard medical services and resources: As an affiliated hospital of Fudan University and a national children's regional medical center, Xiamen Children's Hospital has advanced medical facilities and standardized medical procedures, which ensure the quality and consistency of the medical data collected. In such an environment, the reliability and accuracy of the results are greatly guaranteed. (2) High compliance with follow-up: A team of professional doctors and nurses is responsible for follow-up, which can significantly improve participant compliance. This professional follow-up not only reduces missing data, but also improves the quality and accuracy of data collection. (3) Scientific instrumentation ensures data quality: Critical medical data, such as MRI scans and exome sequencing, are done in a tightly controlled hospital laboratory environment, which avoids the recall bias that comes with questionnaires. (4) Continuous and comprehensive data collection: This cohort is designed at Xiamen Children's Hospital, which can systematically collect children's comprehensive medical data for a long time, including physical examination, treatment records and follow-up information. This mode of data collection provides a solid foundation for research. (5) Lack of selection bias: Due to the specificity of the child population, there is no specific disease in the cohort population, so data collection is not affected by the selection bias that is common in adult cohorts.

### Weakness

(1) Limited extrapolation of the population: Due to the ethnic distribution of the Xiamen population, the main enrolled population consists of Han children, and the results may not be representative of ethnic minorities. This may limit the applicability of the findings to a wider population. (2) Participant loss: A common problem in cohort studies is participant loss over time, and this study cannot avoid loss to follow-up due to moving out.
