# Supplementary material for: Cohort protocol: risk assessment of maternal inflammation and early brain development in infants and young children based on multi-source data modeling
Source: Front Public Health. 2025 Jul 14;13:1530285. doi: 10.3389/fpubh.2025.1530285 (PMC12301305; doi:10.3389/fpubh.2025.1530285)
Supplement: Supplementary file 1 [file Table_1.docx]

# Supplementary

Stable 1 Introduction and brief description of all scales and tasks used in the follow-up process

| Scale/Task Name | Main Content and Purpose | Core Assessment Dimensions |
| --- | --- | --- |
| Griffiths Development Scales - Chinese Edition (GDSC)(1) | Assesses six major development domains of children: 1. Motor ability: covering gross motor and fine motor skills; 2. Personal - social skills include self - feeding, toilet training, and interactions with peers; 3. Language ability: involving vocabulary size, sentence complexity, and non - verbal communication skills; 4. Hand - eye coordination: evaluated through visual - motor integration tasks such as block building and figure tracing; 5. Performance reasoning: examining the ability to solve logical problems like figure matching and sequence sorting; 6. Practical reasoning: assessing the understanding of time concepts and simple mathematical operations. Purpose: Used to identify children with developmental delays, assist in diagnosing neurodevelopmental disorders, and provide baseline data support for early intervention programs. | Motor skills, social skills, language skills, cognitive skills, coordination ability |
| Wechsler Preschool and Primary Scale of Intelligence (WPPSI - IV)(2) | Assesses the intelligence structure through five core indices: 1. Verbal Comprehension: examined through items such as vocabulary definition and similarity reasoning; 2. Visual Spatial: evaluated by tasks like block design and figure assembly; 3. Fluid Reasoning: measured by means of matrix reasoning and picture concept induction; 4. Working Memory: tested with content such as number sequence repetition and picture position memory; 5. Processing Speed: determined by tasks like symbol search and figure cancellation. Purpose: It can identify children with exceptional intelligence or those with intellectual disabilities, analyze the causes of learning difficulties, and thus guide the development of personalized education programs. | Intelligence structure, cognitive strengths and weaknesses |
| Background Questionnaire | Collects information related to family background and children's early experiences: In terms of family structure, it understands whether it is a single - parent or two - parent family and the number of siblings; at the social - economic status level, it covers parental education level, family income, housing conditions, etc.; perinatal information includes pregnancy complications, delivery methods, and birth weight; parenting resources involve early education participation, the number of books and toys at home, etc. Purpose: Serves as a covariate in research to control the impact of environmental factors on children's development. | Demographic variables, early risk factors |
| Index of Child Care Environment (ICCE)(3) | Evaluates the quality of the childcare environment from four major dimensions: 1. Physical safety focuses on the cleanliness of facilities and hazard protection measures; 2. Emotional support: considering the sensitivity of caregivers' responses and the frequency of positive interactions; 3. Cognitive stimulation: assessing the provision of age - appropriate toys, reading activities, and exploration opportunities; 4. Health management: involving dietary nutrition status and disease prevention measures. Purpose: Quantifies the promoting effect of environmental quality on children's development and provides a basis for rating childcare institutions and optimizing relevant policies. | Environmental safety, educational support level |
| Parenting Stress Index-Short Form (PSI-SF)(4) | Measures parental stress from three dimensions: 1. Parental distress: referring to issues such as the deprivation of personal time and financial stress caused by parenting; 2. Dyadic Parent - Child Dysfunction manifests as parents’ perception of emotional alienation or communication difficulties with their children; 3. Difficult Child Characteristics: such as children being irritable, overly dependent, or having other behavioral problems. Purpose: Used to screen high - risk families and provide guidance for parent - child relationship interventions. | Sources of stress, quality of the parent - child relationship |
| Postpartum Experiences Questionnaire (PPQ)(5) | Evaluates postpartum adaptation from four areas: 1. Physical recovery includes wound pain level, fatigue status, and difficulties in breastfeeding; 2. Psychological adaptation: involving mood swings and identity transformation; 3. Social support: covering the degree of spousal participation and the help from relatives and friends; 4. Parenting challenges: such as dealing with infant crying and irregular daily routines. Purpose: Used to identify risk factors for postpartum depression and optimize puerperal care services. | Physical recovery, psychological adaptation, status of support systems |
| Work - Family Conflict Scale(6) | Measures two - way conflict situations: Work-Family conflict, for example, missing family activities due to overtime work; Family-Work conflict, such as the impact of parenting responsibilities on work efficiency. Purpose: Analyzes the sources of stress for working parents and provides a basis for enterprises to develop family - friendly policies. | Time conflicts, role conflicts |
| Pregnancy Life Events Questionnaire (PLQ)(7) | Records the stress events experienced during pregnancy, which are divided into negative events, such as unemployment, serious illness of relatives, and marital crisis; and positive events, such as promotion and family relocation. Purpose: Evaluates the impact mechanism of life events on pregnancy outcomes. | Nature of events, occurrence time, degree of subjective impact |
| Pregnancy Stress Scale (PPS)(8) | Assesses the unique stressors of pregnant women: 1. Concerns about fetal health: such as anxiety caused by abnormal screening results; 2. Fear of childbirth: including anticipation of pain and concerns about medical interventions; 3. Social stress: involving body image evaluation, interruption of career development, etc. Purpose: Identifies pregnant women in a high - stress state and provides them with prenatal psychological counseling or relaxation training. | Stress at the physical, psychological, and social levels |
| Self - Rating Depression Scale (SDS)(9) | Evaluates depressive symptoms through 20 items: emotional symptoms include feelings of depression and worthlessness; physical symptoms involve insomnia and loss of appetite; psychomotor symptoms are manifested as retardation or agitation. Purpose: Can quickly screen for depressive tendencies in the community population and assist in clinical diagnosis. | Severity of depression |
| State - Trait Anxiety Inventory (STAI - Y)(10) | Differentiates between two types of anxiety: State anxiety is the tense emotion triggered by the current situation; Trait anxiety is a stable anxiety tendency. Purpose: Evaluates the effectiveness of anxiety interventions and studies the stress - coping mechanisms of individuals. | Anxiety type, anxiety intensity |
| Pittsburgh Sleep Quality Index (PSQI)(11) | Evaluates 7 sleep dimensions: 1. Subjective sleep quality; 2. Sleep latency; 3. Sleep duration; 4. Sleep efficiency; 5. Sleep disturbances; 6. Use of hypnotic drugs; 7. Daytime dysfunction. Purpose: Used to diagnose insomnia and track the treatment effect of sleep disorders. | Comprehensive sleep quality status |
| Edinburgh Postnatal Depression Scale (EPDS)(12) | Focuses on the core symptoms of postpartum depression through 10 items such as feelings of self - blame, anxiety, and suicidal thoughts. Purpose: Quickly screens for the risk of postpartum depression in obstetric clinics to avoid missed diagnoses. | Risk level of depression |
| Breastfeeding Self - Efficacy Scale (BSES - SF)(13) | Assesses mothers' confidence in breastfeeding: Technical efficacy includes the ability to achieve proper latching and determine whether the baby is full; problem - solving ability, such as dealing with breast engorgement and cracked nipples; social support, that is, the encouragement received from family members and medical staff. Purpose: Predicts the duration of breastfeeding and guides the development of breastfeeding support strategies. | Confidence in breastfeeding, problem - solving ability |
| Maternal - Infant Bonding Scale (MIBS)(14) | Evaluates mothers' emotional experiences towards their infants: Positive bonding is reflected in feelings of intimacy and pleasure; negative bonding is reflected in feelings of rejection, anger, or indifference. Purpose: Identifies whether there are abnormalities in the early mother - infant relationship and provides guidance for psychological interventions. | Strength and quality of emotional bonding |
| Maternal Postnatal Attachment Scale (MPAS)(15) | Evaluates through mothers' self - assessment of interaction behaviors: Frequency of touch, such as the frequency of hugging and kissing the baby; Responsive sensitivity, that is, the ability to soothe a crying baby in a timely manner; Emotional investment, referring to the degree of enjoying the interaction process and paying attention to the baby's needs. Purpose: Evaluates the security of mother - infant attachment and prevents the risk of neglecting or abusing the baby. | Characteristics of attachment behaviors |
| Parenting Styles and Dimensions Questionnaire (PSDQ)(16) | Classifies parenting styles into three types: The Authoritative style is highly responsive and highly demanding; the Authoritarian style is characterized by low responsiveness and high demandingness; the Permissive style is featured by high responsiveness and low demandingness. Purpose: Analyzes the relationship between parenting styles and children's behavioral problems. | Parents' responsiveness, demandingness, and support for children's autonomy |
| Family Assessment Device (FAD)(17) | Evaluates six functions based on the McMaster model: 1. Problem - solving ability; 2. Communication patterns; 3. Rationality of role division; 4. Appropriateness of emotional response; 5. Degree of emotional involvement; 6. Consistency of behavior control. Purpose: Identifies dysfunctional families and points out the direction for family therapy. | Functional status of the family system |
| Co - Parenting Questionnaire(18) | Evaluates the quality of parental collaboration: In terms of responsibility sharing, it focuses on whether the distribution of parenting tasks is fair; Conflict management involves the way of resolving disagreements; Consistency refers to the unity of educational rules. Purpose: Studies the difficulties faced by divorced families in co - parenting and designs co - parenting support plans. | Degree of cooperation, conflict - handling methods, educational consistency |
| Brief Infant Sleep Questionnaire (BISQ)(19) | Evaluates through parental reports: Sleep patterns: number of night awakenings, way of falling asleep; Sleep problems: difficulty falling asleep, circadian rhythm disorders; Influencing factors: feeding methods, sleep environment. Purpose: Screens for infant sleep disorders and provides guidance for sleep training. | Sleep patterns, presence of problem behaviors |
| Chinese Toddler Temperament Scale (CTTS)(20) | For toddlers aged 1 - 3 years, based on the dimensions of infant temperament assessment, two additional dimensions - Persistence and Frustration Tolerance - are specifically added. At the same time, the attention - related indicators are optimized and adjusted to better suit the characteristics of toddlers at this age. Through the assessment of these dimensions, a more comprehensive understanding of toddlers' temperament characteristics can be achieved. The purpose is to deeply analyze the internal relationship between toddlers' temperament and behavioral problems, provide strong support for early intervention, help parents and professionals better understand toddlers' behaviors, and develop appropriate guiding strategies. | Temperament characteristics and the regulatory relationship between temperament and behavior |
| Ages & Stages Questionnaires: Social - Emotional (ASQ:SE - 2)(21) | Screens children's social - emotional development through a series of questions, mainly focusing on three aspects: emotional regulation, social interaction, and self - care ability. Emotional regulation: observes children’s self - soothing ability; in social interaction, it pays attention to their behaviors such as responding to others' smiles and sharing toys; in self - care ability, it examines children's ability to dress independently and express their needs. Purpose: Identifies potential social disorders in children at an early stage, so as to make timely referrals for professional assessment and provide early support for children's social - emotional development. | Social - emotional abilities, including emotional regulation ability, social interaction ability, and self - care ability |
| Putonghua Communicative Development Inventory (PCDI) - Words and Gestures Version(22) | Assesses the pre - language communication abilities of infants aged 8 - 16 months through parental reports. Specifically, it covers vocabulary understanding (infants’ responses to the names of common items), gesture use (such as pointing and waving goodbye), and vocalization ability (including imitating voices and making conscious sounds). Purpose: Screens for possible language development delays in infants, thereby providing a basis for the development of early language stimulation programs and facilitating the good development of infants' language abilities. | Pre - language communication abilities, such as vocabulary understanding ability, gesture - using ability, and vocalization ability |
| Chinese Communicative Development Inventories (CCDI) - Words and Sentences Version(23) | This scale is mainly used to evaluate the language development milestones of toddlers aged 16 - 30 months. It focuses on the vocabulary spurt period (when toddlers' vocabulary reaches 50 - 100 words), two - word combinations (such as 'Mom hug'), and simple syntax (such as the use of subject - predicate structures). Purpose: Track the language development trajectory of toddlers and Study the impact of a bilingual environment on toddlers' language acquisition, and provides references for toddler language development research and education. | Language complexity, such as vocabulary growth, vocabulary combination ability, and grammatical development, that is, the mastery of simple syntax |
| Child Behavior Checklist (CBCL)(24) | This scale is used to evaluate the internalizing and externalizing problem behaviors of children and adolescents aged 1.5 - 18 years. Internalizing problems mainly include manifestations such as anxiety, depression, and withdrawal. Externalizing problems cover behaviors such as aggression, defiance, and hyperactivity. In addition, there is a DSM - oriented scale to assist in diagnosing problems such as ADHD (Attention - Deficit/Hyperactivity Disorder) and Oppositional Defiant Disorder. Purpose: Assist professionals in diagnosing the behavioral disorders of children and adolescents and evaluating the intervention effect, and adjust the intervention measures in a timely manner. | Types of behavioral problems, divided into internalizing and externalizing problems, and the severity of problems |
| M - CHAT - R/F Modified Checklist for Autism in Toddlers, Revised with Follow - up(25) | This test adopts a two - stage screening model. The initial screening is a 20 - item parental report for toddlers aged 16 - 30 months; then a follow - up interview is conducted to reduce false - positive results. Purpose: Enable the early identification of autism spectrum disorders in toddlers during routine pediatric check - ups, and enables early intervention, thus improving the quality of life and development potential of children with autism. | Social - communication abilities (such as responses to others and eye contact) and the presence of stereotypical behaviors (such as repetitive movements and narrow interests) |
| Seven-in-Seven Screen Exposure Questionnaire(26) | The "Seven-in-Seven Screen Exposure Questionnaire" is applicable to preschool children aged 2 - 6 years. It measures seven items, including daily screen time, the frequency of co - viewing with parents, screen use restrictions and compliance, screen exposure during meals and before bedtime, the age of first screen exposure, and low - quality content exposure. This questionnaire is used to assess children's problematic screen exposure. The total score is divided into two categories: low (<7 points) and high (≥ 7 points). It can also investigate relevant influencing factors and provides references for the formulation of relevant strategies and the monitoring of their effectiveness. | Screen exposure frequency, reflected by usage duration; content quality, judged by content type |
| Theory of Mind (ToM) Tasks(27) | Classic task examples: In the false - belief task, a situation is set up where Sally puts a toy in a basket and then leaves, and Anne moves the toy to a box. The subject is asked "Where will Sally look for the toy when she comes back?" This is to examine whether children can understand that others have beliefs different from their own. In the emotion - recognition task, children are required to identify the emotions of people in photos. By identifying different emotion photos, children's understanding of others' emotional states can be evaluated.  Purpose: Evaluate children's ability to understand others' mental states. It can be an important auxiliary means for diagnosing social disorders such as autism. It helps to understand children's social - cognitive development level and provides a basis for targeted interventions. | Ability to reason about mental states, such as understanding and judging others’ beliefs, emotions, and other mental states |
| Head - Toes - Knees - Shoulders (HTKS) Task(28) | Task rules: In the first stage, children are required to touch their heads when they hear the instruction "touch your head" and touch their feet when they hear "touch your feet". In the second stage, the instructions are reversed, that is, they touch their feet when they hear "touch your head" and touch their heads when they hear "touch your feet". Researchers design the task by observing children's performance under different instruction requirements.  Purpose: Measure the core components of executive function - inhibitory control and working memory. Inhibitory control reflects whether children can suppress their original, automatic responses and act according to new rules. Working memory reflects children's ability to store and process instruction information during the task. The results of this task can be used to predict children's school readiness, because good executive function is crucial for aspects such as concentration and task switching in the learning process. | Inhibitory control ability: reflects the control level of one’s own behavioral responses; Cognitive flexibility: embodies the ability to adjust behaviors according to rule changes |
| Emotional Stroop Task(29) | Experimental design: Emotional words (such as "happy", "sad", etc.) are presented in different colors, and subjects are required to ignore the word meaning and quickly report the color. For example, when seeing the word "sad" displayed in red, the subject needs to say "red". In this process, since the semantics of emotional words interfere with the subject's judgment of color, the degree of interference of emotional information on attention is measured by observing the subject's reaction time and error rate. Purpose: Reveal the individual's emotional regulation mechanism, to understand the ability of individuals to suppress the interference of irrelevant information and maintain attention concentration when facing emotional stimuli. This ability plays an important role in individual emotion management, social interaction, and cognitive activities. This task can be used to study the relationship between emotion and cognition and evaluate psychological traits and abilities related to emotional regulation. | Emotional attention bias: the degree to which an individual’s attention is affected by emotional information; Inhibitory function: reflects the ability to suppress the interference of task - irrelevant emotional information |
| Still Face Experiment(30) | Experimental Process: 1. The mother interacts normally with the baby, creating a warm and positive interaction atmosphere to make the baby familiar with the interaction pattern. 2. The mother suddenly keeps a still face and interrupts the normal interaction. At this time, observe the baby's reactions, such as whether it stares at the mother, makes sounds, or shows changes in movements. 3. Resume normal interaction and observe the baby's reactions again, including emotional changes and the restoration of interaction enthusiasm. Purpose: By observing the baby's coping strategies during social interaction interruptions, assesses the baby's attachment security and emotional regulation ability. Securely attached babies will show obvious unease when the mother has a still face, and can quickly adjust their emotions and resume positive interaction when the mother resumes interaction. Insecurely attached babies may show different reaction patterns such as excessive anxiety, avoidance, or neglect. The results of this experiment can help judge the baby's early emotional development and the quality of the parent - child relationship. | Social referencing ability, which means the baby's ability to observe and interpret the emotional and behavioral cues of others and adjust its own behavior accordingly; Stress response, reflecting the baby's emotional and behavioral response patterns when facing sudden changes in social situations |
| Mother and Baby Interaction(31) | This task tests for verbal expression and social emotion. This semi - structured observation records 10 minutes of mother - child interaction. During the observation, parents are encouraged to interact with their children in a normal way. This observational experiment can reflect a number of indicators, such as maternal sensitivity, infant social referencing, dyadic contingent responses, etc. | Verbal expression and social emotion |
| Strange Situation Test(32) | Main Content: This is a classic experiment designed by Mary Ainsworth. Observe the baby's reactions to separation and reunion through 8 standardized stages: Introduction of a stranger: When the mother is present, a stranger enters the room. Observe the baby's reaction to the stranger. First separation: The mother leaves the room, and the baby is alone with the stranger. Record the baby’s emotional and behavioral performance at this time while observing. First reunion: The mother returns. Observe how the baby responds to the mother's comfort and the speed of emotional recovery. Second separation: The mother leaves again, and the baby is completely alone. Further observe the baby's reaction. Stranger’s return: The stranger tries to comfort the baby. Observe the baby's acceptance of the stranger. Second reunion: The mother returns again and interacts with the baby. Observe the baby's behavior and emotional state at this time.  Purpose: Classify the attachment types of babies according to their behaviors into four categories: Secure type: Babies will show certain unease when the mother leaves but will actively seek comfort when the mother returns and can quickly calm down. Avoidant type: Babies show avoidance of contact when the mother leaves and returns and show relatively distant emotions. Ambivalent type: Babies are extremely uneasy when the mother leaves, and when the mother returns, they show both a desire for contact and angry and resistant emotions. Disorganized type: The baby's behavior shows contradictions or no purpose and lacks clear coping strategies when facing separation and reunion. This test is used to evaluate the attachment security of babies and is of great significance for understanding the baby's early emotional development and the quality of the parent - child relationship. | Separation anxiety response, reunion behavior strategies, and attachment type classification |
| Frustration Tolerance(33) + Delay of Gratification Task(34) | Main Content: Combine two classic paradigms to assess children's emotional regulation and self - control abilities: 1.Frustration tolerance task: Design tasks that cannot be solved immediately. For example, let toddlers try to build complex blocks with incomplete materials, and observe the children's emotional reactions when facing difficulties, such as whether they cry, whether they get angry, and whether their emotions recover and how long it takes. 2.Delay of gratification task: Adopt a variant of Walter Mischel's'marshmallow experiment'. Give children a choice between getting a small reward immediately and waiting for a while to get a larger reward and record the children's waiting time and the self - regulation strategies they use during the waiting process, such as self - comfort and distraction. Purpose: Predict children's future executive function development, social adaptability, and academic achievements. Children who can better delay gratification and maintain emotional stability and respond positively in frustrating situations tend to perform better in future learning and social interactions. This task can also be used to identify the risk of children's self - regulation deficiencies and provide directions for early intervention. | Emotional regulation strategies (such as self - soothing), impulse control ability, goal - persistence, delay choice, and waiting behavior |

**Reference**

1. Li PY, Fu NN, Li QY, Wang GF, Gao L, Zhang X. The Griffiths Development Scales-Chinese (GDS-C): A reliable and valid neurodevelopmental assessment tool in children with ASD aged 3-8 years old in Tianjin, China. Asian J Psychiatr. 2020;52:102144.http://dx.doi.org/10.1016/j.ajp.2020.102144

2. Park SE, Demakis GJ. Wechsler Preschool and Primary Scale of Intelligence. Springer International Publishing. 2017

3. Anme T, Tanaka E, Watanabe T, Tomisaki E, Y M, Tokutake K. Validity and Reliability of the Index of Child Care Environment (ICCE). Public Health Frontier. 2013.http://dx.doi.org/10.5963/PHF0203003

4. Luo J, Wang MC, Gao Y, Zeng H, Yang W, Chen W, et al. Refining the Parenting Stress Index-Short Form (PSI-SF) in Chinese Parents. Assessment. 2021;28(2):551-66.http://dx.doi.org/10.1177/1073191119847757

5. Zhang D, Zhang J, Gan Q, Wang Q, Fan N, Zhang R, et al. Validating the Psychometric Characteristics of the Perinatal Posttraumatic Stress Disorder Questionnaire (PPQ) in a Chinese Context. Arch Psychiatr Nurs. 2018;32(1):57-61.http://dx.doi.org/10.1016/j.apnu.2017.09.016

6. Haslam D, Filus A, Morawska A, Sanders M, Fletcher R. The Work–Family Conflict Scale (WAFCS): Development and Initial Validation of a Self-report Measure of Work–Family Conflict for Use with Parents. Child psychiatry and human development. 2014;46.http://dx.doi.org/10.1007/s10578-014-0476-0

7. Lu Z, Jia-hu HAO, Ling T, et al. Compilation and item selection of a pregnancy life events questionnaire. Chinese Journal of Public Health. 2015;31(8):1026-9.http://dx.doi.org/10.11847/zgggws2015-31-08-13

8. Tang X, Lu Z, Hu D, Zhong X. Influencing factors for prenatal Stress, anxiety and depression in early pregnancy among women in Chongqing, China. J Affect Disord. 2019;253:292-302.http://dx.doi.org/10.1016/j.jad.2019.05.003

9. Thurber S, Snow M, Honts CR. The Zung Self-Rating Depression Scale: convergent validity and diagnostic discrimination. Assessment. 2002;9(4):401-5.http://dx.doi.org/10.1177/1073191102238471

10. Valente G, Diotaiuti P, Corrado S, Tosti B, Zanon A, Mancone S. Validity and measurement invariance of abbreviated scales of the State-Trait Anxiety Inventory (STAI-Y) in a population of Italian young adults. Front Psychol. 2025;16:1443375.http://dx.doi.org/10.3389/fpsyg.2025.1443375

11. Buysse DJ, Reynolds CF, 3rd, Monk TH, Berman SR, Kupfer DJ. The Pittsburgh Sleep Quality Index: a new instrument for psychiatric practice and research. Psychiatry Res. 1989;28(2):193-213.http://dx.doi.org/10.1016/0165-1781(89)90047-4

12. Cox JL, Holden JM, Sagovsky R. Detection of postnatal depression. Development of the 10-item Edinburgh Postnatal Depression Scale. Br J Psychiatry. 1987;150:782-6.http://dx.doi.org/10.1192/bjp.150.6.782

13. Amini P, Omani-Samani R, Sepidarkish M, Almasi-Hashiani A, Hosseini M, Maroufizadeh S. The Breastfeeding Self-Efficacy Scale-Short Form (BSES-SF): a validation study in Iranian mothers. BMC Res Notes. 2019;12(1):622.http://dx.doi.org/10.1186/s13104-019-4656-7

14. Bienfait M, Maury M, Haquet A, Faillie JL, Franc N, Combes C, et al. Pertinence of the self-report mother-to-infant bonding scale in the neonatal unit of a maternity ward. Early Hum Dev. 2011;87(4):281-7.http://dx.doi.org/10.1016/j.earlhumdev.2011.01.031

15. Makeen M, Farrell LM, LaSorda KR, Deng Y, Altamirano V, Jarvis O, et al. Associations between postpartum pain, mood, and maternal-infant attachment and parenting outcomes. Sci Rep. 2022;12(1):17814.http://dx.doi.org/10.1038/s41598-022-21793-1

16. Robinson C, Mandleco B, Roper S, Hart C. The Parenting Styles and Dimensions Questionnaire (PSDQ). Handbook of Family Measurement Techniques. 2001;3:319-21

17. Mansfield AK, Keitner GK, Archambault RA. Family Assessment Device. In: Lebow J, Chambers A, Breunlin DC, editors. Encyclopedia of Couple and Family Therapy. Cham: Springer International Publishing; 2018. p. 1-5.

18. Margolin G. Coparenting questionnaire. Unpublished instrument, University of Southern California, Los Angeles. 1992

19. Sadeh A. A brief screening questionnaire for infant sleep problems: validation and findings for an Internet sample. Pediatrics. 2004;113(6):e570-7.http://dx.doi.org/10.1542/peds.113.6.e570

20. Hong Q, Zhou S, Yao K, Liu L. Standardized and revised the toddler temperament scale. Chin J Child Health Case. 1998;6:201-4

21. Squires J, Bricker D, Twombly E. Ages & stages questionnaires: Social-emotional. Baltimore: Brookes. 2002

22. Kessler D. Predictive Reliability of the Mandarin Chinese (Putonghua) Communicative Developmental Inventories Across Early Performance-Based Strata 2009.

23. Yue A, Luo X, Jia M, Wang B, Gao Q, Shi Y, et al. Concurrent validity of the MacArthur communicative development inventory, the Ages and Stages Questionnaires and the Bayley Scales of Infant and Toddler Development: A study in rural China. Infant and Child Development. 2021;30(3):e2219, 1522-7227

24. Kristensen S, Henriksen TB, Bilenberg N. The Child Behavior Checklist for Ages 1.5–5 (CBCL/1½–5): Assessment and analysis of parent-and caregiver-reported problems in a population-based sample of Danish preschool children. Nordic journal of psychiatry. 2010;64(3):203-9, 0803-9488

25. Robins DL, Casagrande K, Barton M, Chen CM, Dumont-Mathieu T, Fein D. Validation of the modified checklist for Autism in toddlers, revised with follow-up (M-CHAT-R/F). Pediatrics. 2014;133(1):37-45.http://dx.doi.org/10.1542/peds.2013-1813

26. Yalcin SS, Tezol O, Caylan N, Erat Nergiz M, Yildiz D, Cicek S, et al. Evaluation of problematic screen exposure in pre-schoolers using a unique tool called "seven-in-seven screen exposure questionnaire": cross-sectional study. BMC Pediatr. 2021;21(1):472.http://dx.doi.org/10.1186/s12887-021-02939-y

27. Carlson SM, Koenig MA, Harms MB. Theory of mind. Wiley Interdisciplinary Reviews: Cognitive Science. 2013;4(4):391-402, 1939-5078

28. McClelland MM, Cameron CE, Duncan R, Bowles RP, Acock AC, Miao A, et al. Predictors of early growth in academic achievement: The head-toes-knees-shoulders task. Frontiers in psychology. 2014;5:599, 1664-078

29. Williams JMG, Mathews A, MacLeod C. The emotional Stroop task and psychopathology. Psychological bulletin. 1996;120(1):3, 1939-455

30. Adamson LB, Frick JE. The still face: A history of a shared experimental paradigm. Infancy. 2003;4(4):451-73, 1525-0008

31. Chung FF, Wan GH, Kuo SC, Lin KC, Liu HE. Mother-infant interaction quality and sense of parenting competence at six months postpartum for first-time mothers in Taiwan: a multiple time series design. BMC Pregnancy Childbirth. 2018;18(1):365.http://dx.doi.org/10.1186/s12884-018-1979-7

32. Simonelli A, Parolin M. Strange situation test. Encyclopedia of Personality and Individual Differences Cham, Switzerland: Springer. 2016

33. Meindl P, Yu A, Galla BM, Quirk A, Haeck C, Goyer JP, et al. A brief behavioral measure of frustration tolerance predicts academic achievement immediately and two years later. Emotion. 2019;19(6):1081, 931-516

34. Duckworth AL, Tsukayama E, Kirby TA. Is it really self-control? Examining the predictive power of the delay of gratification task. Personality and Social Psychology Bulletin. 2013;39(7):843-55, 0146-1672
